# Leakage of old carbon dioxide from a major river system in the Canadian Arctic

**DOI:** 10.1093/pnasnexus/pgae134

**Published:** 2024-03-29

**Authors:** Sanjeev Dasari, Mark H Garnett, Robert G Hilton

**Affiliations:** Department of Earth Sciences, University of Oxford, Oxford OX1 3AN, UK; NEIF Radiocarbon Laboratory, SUERC, Rankine Avenue, East Kilbride G75 0QF, UK; Department of Earth Sciences, University of Oxford, Oxford OX1 3AN, UK

**Keywords:** dissolved inorganic carbon, carbon isotopes, permafrost, Arctic carbon cycle, CO_2_ evasion

## Abstract

The Canadian Arctic is warming at an unprecedented rate. Warming-induced permafrost thaw can lead to mobilization of aged carbon from stores in soils and rocks. Tracking the carbon pools supplied to surrounding river networks provides insight on pathways and processes of greenhouse gas release. Here, we investigated the dual-carbon isotopic characteristics of the dissolved inorganic carbon (DIC) pool in the main stem and tributaries of the Mackenzie River system. The radiocarbon (^14^C) activity of DIC shows export of “old” carbon (2,380 ± 1,040 ^14^C years BP on average) occurred during summer in sampling years. The stable isotope composition of river DIC implicates degassing of aged carbon as CO_2_ from riverine tributaries during transport to the delta; however, information on potential drivers and fluxes are still lacking. Accounting for stable isotope fractionation during CO_2_ loss, we show that a large proportion of this aged carbon (60 ± 10%) may have been sourced from biospheric organic carbon oxidation, with other inputs from carbonate weathering pathways and atmospheric exchange. The findings highlight hydrologically connected waters as viable pathways for mobilization of aged carbon pools from Arctic permafrost soils.

**Significance Statement**

Warming of the circumpolar north can lead to the release of carbon stocks from thawing of organic-rich permafrost soils and enhanced chemical weathering of rocks. Here, by tracing the isotopic fingerprints of dissolved inorganic carbon pool in a northern Canadian Arctic River system, we show that transfer of old C occurred on two separate occasions. The evading CO_2_ in the river system is plausibly sourced from the oxidation of aged organic matter, representing a leak of carbon from millennial terrestrial C stocks through hydrologically connected waters. Such C mobilizing pathways are of concern for future climate warming of permafrost zones. There is therefore an urgent need to better understand the drivers of greenhouse gas release in such climatically vulnerable regions.

## Introduction

**Fig. 1.**
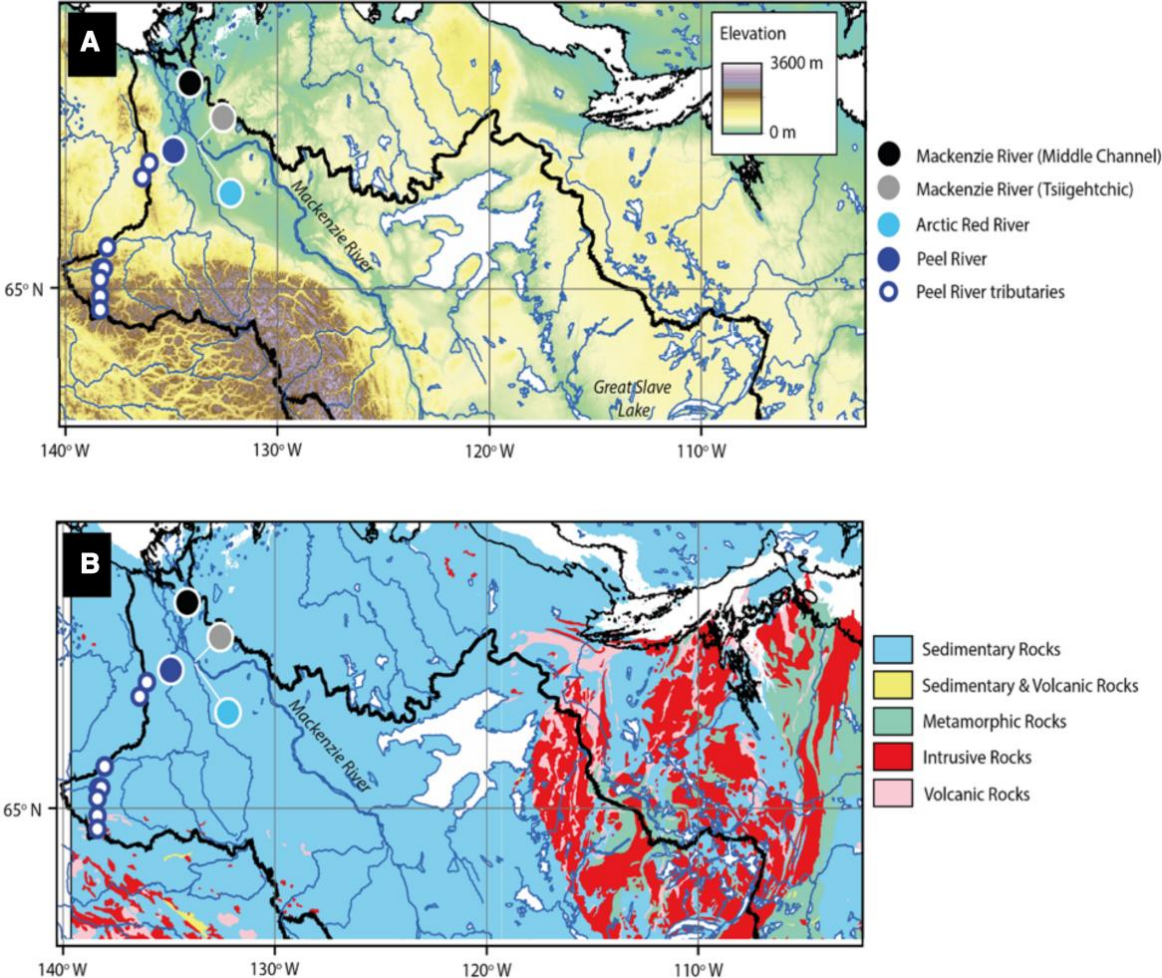
The Mackenzie River Basin and sampling locations of this study. A) Elevation (GDEM 30 arc second) shown with river sampling locations. B) Bedrock geology of the sampling area, coded by major rock type (20). Continuous permafrost dominates these locations (21).

Accelerated rates of climate warming in the high latitudes are likely to induce acute changes in the major components of the Arctic cryosphere (1, 2). In northern Canada, the annual average air temperature has increased 2.3 °C over the period of 1948–2016 and is projected to increase 7.8 ° C by 2081–2100 relative to the period 1986–2005 (3). This could impact land-to-ocean transfer of biogeochemical components, with implications for the environment, ecosystem, and broad-scale Arctic biogeochemical cycling. One such component, the Arctic carbon cycle, is prone to various processes influenced by warming (4). In the circumpolar north, peat deposits and deltaic sediments have accumulated organic-rich permafrost soils over millennia (5–7). Warming-induced permafrost thaw and subsequently increased soil respiration could eventually lead to the release of greenhouse gases (e.g. CO_2_, CH_4_) through microbial decomposition and/or photodegradation, thereby exacerbating future climate change (8, 9). Changes in hydrology and the nature of mineral–water interactions following permafrost thaw, alongside warming-induced changes to plant distribution and physiology, could also contribute to enhanced chemical weathering and rock carbon inputs, which transfer carbon on timescales of 10^4^–10^6^ years (10, 11). Both these processes (i.e. old soil respiration and chemical weathering) could potentially be active in certain northern landscapes, e.g. (12). Tracking the age and fate of the carbon pool in river systems remains a priority for future projections of Arctic warming and climate change. Currently, process-level understanding remains elusive and is hampered by the challenges of sample collection from remote locations (13–15). Rivers, dubbed as “biogeochemical reactors”, integrate the characteristics of the surrounding landscapes, and are not exempt from the impacts of global warming and climate change (16). As such, it is possible to track vital information about C-dynamics from the basin-specific characteristics of the organic and inorganic carbon pools, including particulate organic carbon (POC) and dissolved organic carbon (DOC) and dissolved inorganic carbon (DIC), e.g. (12, 17–19). In particular, the application of radiocarbon has shed new light on the age, source, and pathways of carbon in these pools, e.g. (18, 19). Here, we focus on the Mackenzie River Basin and its associated tributaries (total drainage area 1.8 × 10^6^ km^2^) as the largest contributor of freshwater and DIC and second largest contributor of DOC, and as such a major carbon source from the North American continent to the Arctic Ocean (12, 18). The Mackenzie River Basin exhibits varied soil organic matter content and is dominated by sedimentary rock lithologies, with half of the basin lying within permafrost covered zones (Fig. 1). Two of its large tributaries (Arctic Red: 21.8 × 10^3^ km^2^; Peel River: 70.6 × 10^3^ km^2^) almost exclusively drain continuous permafrost (12). Several works exist on the sources and fluxes of DOC in Arctic rivers, justified by its link to changing hydrological pathways and its potential reactivity, e.g. (19, 22, 23). In the Mackenzie River, analysis of long-term records in the main stem has established increasing DOC (and DIC) fluxes in the past decades (12), while radiocarbon (^14^C) activity of DOC suggests a mostly “modern” origin, implying that vegetation and soil derived, rapidly cycling, young organic matter likely dominates DOC in this river basin (18, 23). This contrasts starkly with the POC pool, which has a ^14^C composition reflecting erosion of aged soil organic matter and rock organic carbon inputs (18, 19, 23). In stark contrast, there are no measurements of the age of one of the major carbon pools, DIC, in this river basin, reflecting a broader deficit of radiocarbon DIC measurements in the northern latitudes (>60°N) (24, 25). We aim to address this major knowledge gap to better understand the riverine C-export in one of the world's most climatically vulnerable regions. The DIC pool is influenced by a mixture of sources and processes. Chemical weathering of carbonate and silicate minerals by carbonic acid contribute importantly to the DIC pool (11, 26, 27), with DIC mostly in the form of bicarbonate (HCO_3_^−^) whose production represents a CO_2_ sink [Eq (1–2)]. In the Mackenzie River Basin and other locations where sedimentary rocks dominate the lithology (Fig. 1), sulfide mineral (e.g. pyrite) oxidation is a pathway for carbonate dissolution, potentially contributing to the present day HCO_3_^−^ flux, (Eq. 3) (12, 26–28), or this CO_2_ may be released to the atmosphere at the reaction site (29)


(1) $$\mathrm{CaSi}O_{3}+2CO_{2}+H_{2}O\to Ca^{2+}+\mathrm{Si}O_{2}+2\mathrm{HC}O_{3}^{-}$$

(2) $$\mathrm{CaMg}(CO_{3}{)}_{2}+2CO_{2}+2H_{2}O\to Ca^{2+}+Mg^{2+}+4\mathrm{HC}O_{3}^{-}$$

(3) $$2\mathrm{CaC}O_{3}+H_{2}SO_{4}\to 2Ca^{2+}+SO_{4}^{2-}+2\mathrm{HC}O_{3}^{-}\to Ca^{2+}+SO_{4}^{2-}+\mathrm{CaC}O_{3}+CO_{2}+2H_{2}O$$

**Fig. 2.**
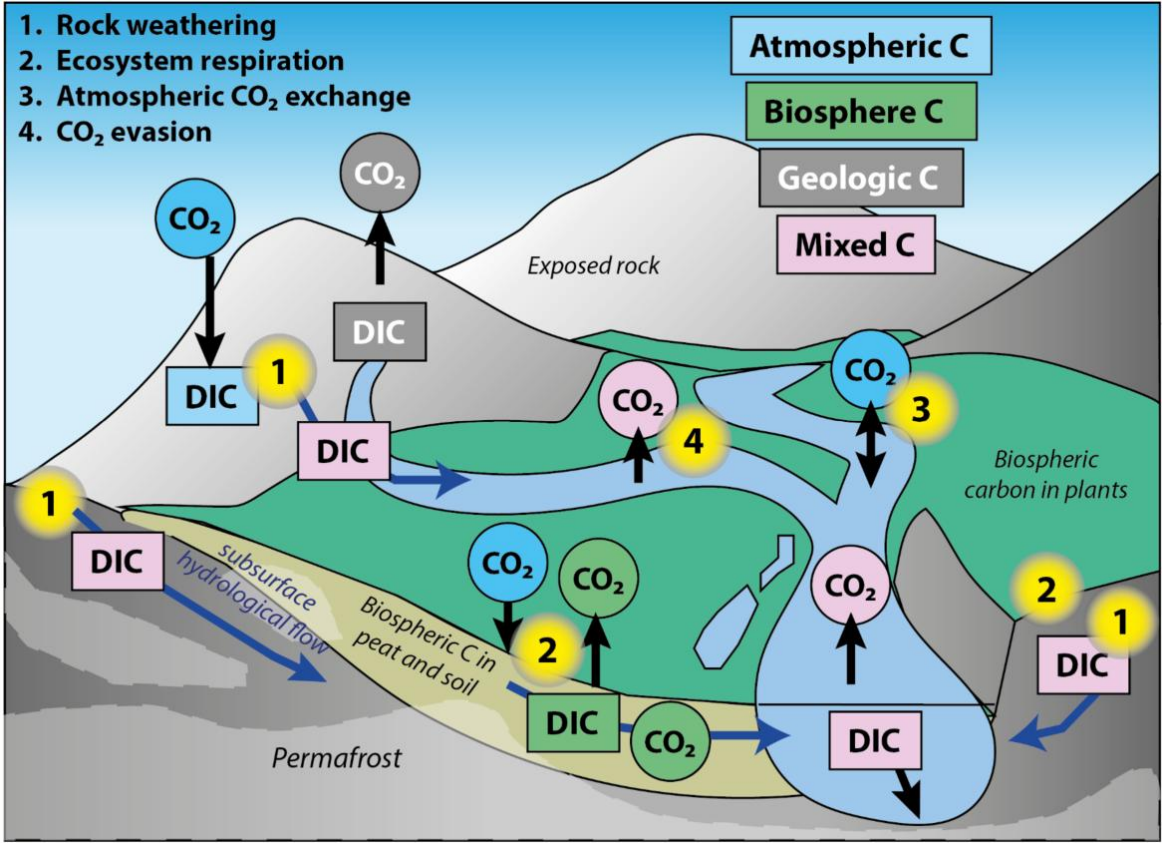
The controls on DIC isotope composition in the Mackenzie River Basin. Pathways of atmospheric CO_2_, C from the vegetation and soil, C from rock organic matter and carbonate minerals and mixtures of these sources are conceptualized. Permafrost present in bedrock and peat and soil is shown. DIC can be produced by rock weathering (1), introducing atmospheric and geological C into the river DIC pool. Ecosystem respiration produces CO_2_ (2), and this can contribute to the DIC pool in the landscape, and sources 1 + 2 can be moved by hydrological pathways to the river network. Atmospheric exchange may act to modify the DIC isotope composition (3), while CO_2_ evasion from river surfaces (4) will impact the stable isotope composition.

However, this weathering-derived bicarbonate is not conservative, and there could be large influxes of CO_2_ into the DIC pool from temperature-dependent decomposition of soil organic matter (28, 30) or within river heterotrophic respiration (16). In some less turbid rivers, uptake of CO_2_ by aquatic photosynthesis may also be important (31). In terms of inputs from the catchment, the CO_2_ produced in near-surface soil includes autotrophic respiration of rapidly cycling carbon as well as heterotrophic decomposition of carbon that cycles on a broad range of timescales (30). In the lower depths of the seasonally thawed active layer, however, carbon that has cycled on millennial timescales may decompose under aerobic and thawed conditions and seep into the interconnected hydrological network (13, 14, 19). As such, it is expected that both “modern” and “aged” CO_2_ are exchanging C within the Mackenzie River DIC pool, respectively (Fig. 2). Most river systems have been found to be supersaturated in dissolved CO_2_ relative to the atmosphere, meaning they act as a large global source (32). With such diverse sources of riverine DIC (and CO_2_) in this river system, it is challenging to resolve the potential drivers using only source markers or conservative tracers. Dual-carbon isotopic probing using stable carbon isotope (δ^13^C) and radiocarbon (Δ^14^C) may contain information attributable to DIC source identification, e.g. (28, 33). In the past, such measurements have also provided valuable information on the residence times, transformations, and interactions of other carbon reservoirs (34–36). Therefore, we collected DIC samples for δ^13^C and Δ^14^C from the Mackenzie River mainstem and its northern tributaries, the Peel and Arctic Red rivers (Fig. 1). The samples in the present study can be placed in the context of the large existing body of work on the river POC (18, 23) and DOC (17, 19) alongside constraints on chemical weathering (11, 12, 26, 27), allowing us to investigate the origin of DIC in this high-latitude Arctic river network and the implications for CO_2_ released from river surfaces.

## Results

**Fig. 3.**
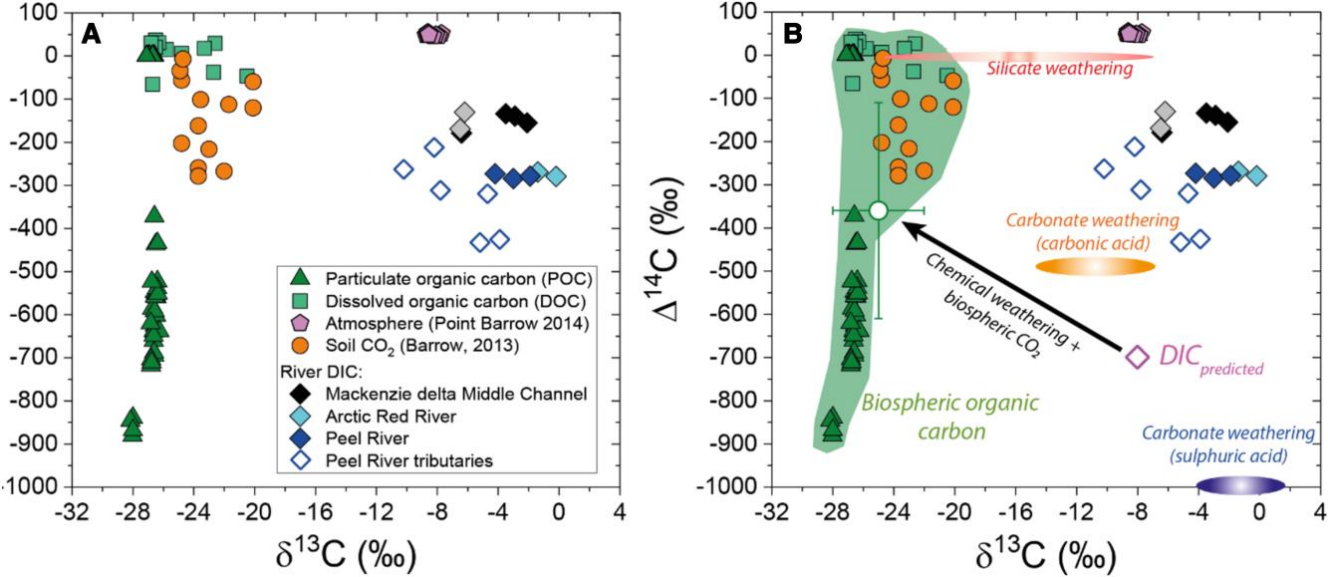
Dual isotope characteristics of DIC in the Mackenzie River system. A) New river DIC measurements (diamonds; “black filled”—Mackenzie River Middle Channel, “gray filled”—Mackenzie River at Tsiigehtchic, “cyan filled”—Arctic Red River, “blue filled”—Peel River, “open”—Peel River Tributaries) are shown alongside measurements that characterize potential sources of carbon to the DIC pool which include river POC (triangle) (18), DOC (squares) (17), soil CO_2_ (circles) (37). and atmospheric CO_2_ (pentagons) from Point Barrow (38). B) The range of carbon compositions that capture biospheric organic matter oxidation are shaded green, alongside an inferred end member and SD on compositions (circle, Table S3). The endmember compositions of weathering are shown. DIC_predicted_ is the result of the previously quantified chemical weathering fluxes (26, 27) coupled to their isotopic endmembers (mixing components shown as gray dotted line, Table S4). The expected trajectory for a two-component mixing (chemical weathering and biosphere organic carbon oxidation) is shown (black line).

The DIC concentrations, δ^13^C_DIC_ and Δ^14^C_DIC_ values ranged between 12 and 21 mg/L, −6.4‰ and −2.1‰, and −179‰ and −134‰ in the Mackenzie River samples from upstream of the delta (Tsiigehtchic) and in the Middle Channel of the delta, respectively. In comparison, the Arctic Red River, Peel River and Peel River tributaries, the DIC concentrations, δ^13^C_DIC_ and Δ^14^C_DIC_ values ranged between 9 and 33 mg/L, −10.2‰ and −0.2‰, and −434‰ and −130‰, respectively (Fig. 3; Tables S1 and S2). On average, the observed Δ^14^C_DIC_ in the Mackenzie River system (−250 ± 93‰, with SD range, *n* = 17 Fig. 3) was found to be much lower than the reported Δ^14^C_DIC_ in e.g. streams (−112 ± 113‰) (33), tropical rivers (43 ± 73‰) (28), and sea and ocean surfaces (−79 ± 108‰) (36). Here, a characteristic difference is the presence of aged DIC, with the radiocarbon ages ranging between 1,060 and 4,492 ^14^C years BP (before present). To the best of our knowledge, such DIC ages have not been reported for any large northern river system to date (34).

The observed δ^13^C_DIC_ in the Mackenzie River system (−5 ± 3 ‰) was enriched yet comparable to the reported δ^13^C_DIC_ in e.g. tropical rivers (−11 ± 2 ‰) (28) and streams (−8 ± 1 ‰) (33). The δ^13^C_DIC_ values appear to overlap with the isotope end members for chemical weathering processes (Tables S1–S3). Decomposition of organic matter within river systems will also contribute to riverine DIC, and hence alter DIC isotopic ratios. In such a scenario, the δ^13^C_DIC_ from within-system organic matter remineralization will be similar to the δ^13^C signature of the riverine organic matter which reflects a mixture of C_3_ biomass dominated inputs (−25 ± 3‰; see POC and DOC in Table S3) (18, 19, 28, 37). In comparison, the observed δ^13^C_DIC_ values in the Mackenzie River system are nearly ∼20‰ enriched overall. This could result from the predominance of a single DIC source with distinct δ^13^C signature, e.g. carbonate weathering by sulfuric acid. Such a possibility exists given the pronounced multi-decadal increase in sulfate flux of ∼ 64% in this system (12). However, processes such as atmospheric exchange (i.e. with soils and water) also fractionate δ^13^C and could result in shifts in δ^13^C_DIC_ during riverine transport (39–43), which we discuss in the next section.

**Fig. 4.**
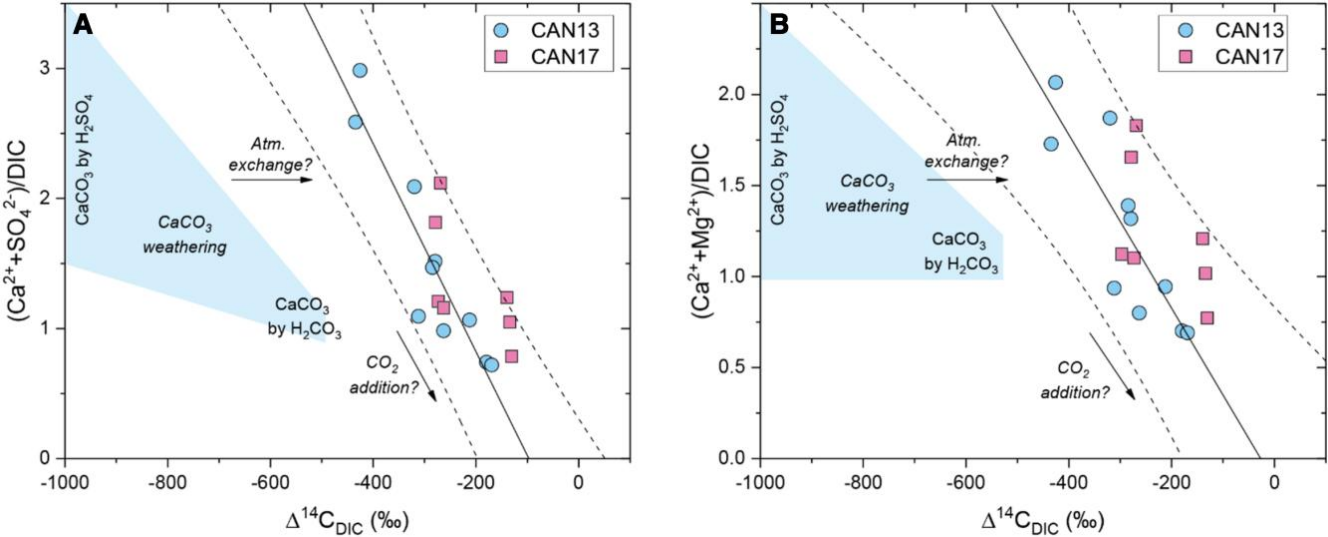
The influence of chemical weathering on the Mackenzie River DIC radiocarbon content. The ratio of the weathering products from carbonate and sulfide mineral weathering, i.e. water-soluble ion concentrations, are shown normalized to DIC concentrations, along with the radiocarbon content of the DIC for: A) calcium and sulfate ions and B) calcium and magnesium ions. Blue shaded region denotes zone of compositions produced by theoretical carbonate weathering reactions by carbonic (H_2_CO_3_) and sulfuric acid (H_2_SO_4_). Samples lie outside this domain and suggest the potential role of both isotopic exchange with atmospheric CO_2_ and addition of DIC with a higher Δ^14^C value than weathering inputs. Linear fits and 95% confidence interval are shown as dashed lines and suggest addition of DIC while Δ^14^C increases.

The major ion concentrations ([Ca^2+^], [Mg^2+^], and [SO_4_^2−^]; Table S1) were comparable with previously reported values in this river system (12). The observed values were highest in the Peel River samples and lowest in the Mackenzie mainstem, which supports previous work highlighting high rates of carbonate and sulfide mineral weathering in the Peel River (12, 26, 27) (Fig. 1B). The ratios of these ions and DIC are used to track the relative importance of chemical weathering. In particular, we note a significant positive correlation between weathering components (Ca^2+^, Mg^2+^, SO_4_^2−^) and Δ^14^C_DIC_ (*R*^2^ > 0.9, *P* < 0.001; Fig. 4, see also Fig. S1).

## Discussion

The averaged Δ^14^C_DIC_ values in global large rivers have been found to be −32 ± 140‰ (34). The aged DIC we have observed in the Mackenzie River system (Δ^14^C_DIC_ = −250 ± 93‰, or 2,380 ± 1,040 ^14^C years BP on average) has not been documented previously in any large Arctic rivers (17, 34). To explain the aged DIC, here we consider the DIC and CO_2_ inputs and losses which may occur throughout this northern landscape (Fig. 2). First, we calculate a “predicted” DIC isotopic composition expected for the known chemical weathering pathways of rocks in the landscape. From this starting point, we then explore how CO_2_ inputs from organic matter respiration and decomposition, either sourced in the landscape and/or within the river water, may contribute to the isotopic composition of the river DIC pool. The loss of CO_2_ from river surfaces (CO_2_ evasion) is also considered, as it can induce stable isotope fractionation, and the role of atmospheric CO_2_ exchange assessed. By considering these factors together, we put forward a hypothesis to explain the Mackenzie River Δ^14^C_DIC_ and δ^13^C_DIC_ compositions, with old carbon source from a combination of carbonate weathering inputs and aged carbon from organic matter degradation.

### Predicted DIC isotopic signatures from chemical weathering processes

Previous work on the Mackenzie River Basin has quantified the fluxes of CO_2_ drawdown and bicarbonate production by carbonate and silicate weathering (11, 26, 27). Weathering of sedimentary rocks dominates the dissolved cation load in this river system (27). Based on mass balance of dissolved weathering products from previous work (11, 26, 27), an estimated 48% of the DIC pool should have been derived from carbonate mineral weathering with carbonic acid, likely derived from atmospheric, soil CO_2_, and potentially from rock organic carbon oxidation (27). An additional 38% of the DIC flux is attributed to carbonate weathering by sulfuric acid (26). A further 14% of DIC is derived from silicate mineral weathering by carbonic acid. Rock organic carbon oxidation has been quantified in this basin, but the fate of the CO_2_ remains uncertain (18). It could enter the DIC pool, or be released directly as atmospheric CO_2_ (29). If entering the DIC pool, this would offset the atmospheric CO_2_ used in carbonic acid weathering of carbonate and silicate minerals (see Table S4). This would equate to 11% of the carbonic acid weathering pool deriving from rock organic carbon oxidation, with the remaining 51% assumed to derive from atmospheric or soil CO_2_. Coupling these contributions to their respective dual-isotopic signatures leads to a predicted DIC isotopic signature (“DIC_predicted_” in Fig. 3; Table S4). The DIC_predicted_ value cannot explain the measured DIC isotopic compositions. The river DIC is much younger than that expected for weathering inputs (Fig. 3). However, a clear imprint of weathering can still be seen on the Δ^14^C_DIC_ by examining the dissolved ion loads. Correlations between carbonate and sulfide mineral weathering products and the DIC radiocarbon activity are found (Fig. 4). The most ^14^C-depleted samples generally have the highest (Ca^2+^ + Mg^2+^)/DIC and (Ca^2+^ + SO_4_^2−^)/DIC values, and these come from the tributaries of the Peel River where carbonate and sulfide weathering are widespread (26). These general patterns (between Δ^14^C_DIC_ and carbonate dissolution) have also been reported from the Amazon River (35) and Tibetan Plateau streams (43). While this suggests an important weathering input to the modern DIC pool of the Mackenzie River system, the DIC ages are not old enough to solely reflect the weathering inputs (e.g. the DIC_predicted_ ∼ 6,000 ^14^C years BP) and there is a large amount of variability in ^14^C age of DIC for a given dissolved geochemical composition (Fig. S1).

To explain the younger river DIC signal than predicted for chemical weathering inputs, we have identified three possibilities. First, some of the CO_2_ from carbonate weathering by sulfuric acid and rock organic matter oxidation may not enter the DIC pool, as suggested by outcrop scale measurements of shale weathering (29, 44). This would act to increase the (Ca^2+^ + Mg^2+^)/DIC but we would not find the carbonate weathering Δ^14^C signature in the DIC pool. Second, we could invoke atmosphere exchange. Isotope exchange between river DIC and atmospheric CO_2_ would act to increase Δ^14^C_DIC_, but would not influence (Ca^2+^ + Mg^2+^)/DIC ratios. While both processes could be acting, they cannot explain the other main feature of the dataset: the negative linear relationships between river Δ^14^C_DIC_ and (Ca^2+^ + Mg^2+^)/DIC and (Ca^2+^ + SO_4_^2−^)/DIC (Fig. 4). These relationships suggest that as DIC increases relative to Ca^2+^ and Mg^2+^, Δ^14^C_DIC_ increases. In other words, DIC enrichment from a carbon source with a “younger” Δ^14^C_DIC_ value could explain the linear trends. Based on regressions to the data, this DIC input appears to have a Δ^14^C value < 0‰ (see *x*-intercept in Fig. 4). Therefore, the third option to explain the geochemical data is that there is an important input of DIC from plausibly biospheric organic carbon (see Fig. 3).

### DIC input from the biospheric organic carbon oxidation

The presence of aged DIC in this high-latitude river system is likely given the large volumes of organic matter in soils, and the pathways of organic carbon transfer from soil to streams (Fig. 2). The oxidation of biospheric organic matter in the landscape could deliver CO_2_ to the river DIC pool via hydrological pathways (45–47). Alternatively, within river processing of a river DOC pool or degradation of eroded organic matter in particulate form (river POC) could add CO_2_ to the river DIC pool.

Previous work has explored the mobilization and age of DOC in the Mackenzie River and found the majority of river DOC is young, with a similar Δ^14^C value to atmospheric CO_2_ in the year of sampling (17, 19) (Fig. 3A). However, a one-time input of aged DOC into the Mackenzie River system was found in June 2018 (19) followed a pronounced warm temperature anomaly in winter 2017/2018 and an anomalous warm summer period in 2017 which was followed by colder summer seasons in 2018 and 2019. During the DIC sampling periods in July 2013 and June 2017, neither a temperature anomaly nor any broad-scale changes in the water discharge were encountered (Fig. S2) and at these times the sampled DOC was Δ^14^C_DOC_ > 0‰ (19). The remineralization or photooxidation of allochthonous DOC could provide the additional DIC input we propose. However, the DIC enrichment appears to have a Δ^14^C_DIC_ value <0‰ (Fig. 4). Interestingly, the contrast between DIC and DOC ^14^C age suggests significant quantities of DIC cannot be the product of autotrophic DOC uptake in the river at these sampling periods (i.e. primary production) (28).

A plausible pathway for DIC inputs is the leakage of CO_2_ from organic-rich permafrost zones carried by modified hydrological pathways (45–47) (Fig. 2). Long-term observations of air temperature records from Inuvik and Norman Wells show temperature increases over the last 76 years during the freezing season (Fig. S2). This influences the dynamics and thickness of the active layer modulated by the seasonally varying temperatures. Indeed, in the northern Mackenzie River Basin, a thickening of the active layer by about 10% has been reported since 2000 (48). The development of thin, perennial taliks within and above permafrost is ensued during the summer months from the vertically and laterally thawing permafrost table. This thaw is sustained further with mild winters (49–51). Increased hydrological connectivity can then enhance drainage of surface soils, and consequently the organic soils can be undersaturated prior to freeze back in fall (52). During spring and in this regime, a larger portion of meltwater can thus infiltrate soils, supplying sensible heat to the soil and leading to the thawing of the upper permafrost table, further expanding the hydrologically connected pathways (14, 45, 52). CO_2_ from soil respiration and decomposition below the active layer can thus be mobilized by this subsurface flow of water. An additional coupled process could be the formation of thermokarst erosion features, which also allows deep, old soil organic matter to be exposed to O_2_-rich atmosphere and waters (14, 47). These mechanisms would result in subsequent transfer of dissolved CO_2_ to the river system where it can exchange with the river DIC pool (28, 37, 46).

### Constraining the biospheric organic carbon oxidation isotopic endmember

To quantify how biospheric organic carbon oxidation may contribute to the DIC pool in the Mackenzie River, we need to establish its radiocarbon and stable isotope composition. The Δ^14^C signatures of CO_2_ from soil respiration and decomposition could have a wide range due to the decomposition of organic carbon pools of varying age (30). Studies on the ^14^C content of respired soil CO_2_ indicate that most soil respiration is from organic matter sources with a Δ^14^C > 0‰, as also observed recently in a similar northern site (28, 30, 37). However, an assessment of the influence of chemical weathering on the Mackenzie River DIC radiocarbon content shows that in a no weathering-C input scenario, the river Δ^14^C_DIC_ still appears to be < 0‰ (see *x*-intercept in Fig. 4), implying that CO_2_ from recent ecosystem respiration (Fig. 3) may not be a viable source in this case. Soil pore CO_2_ can contribute to riverine DIC. The Δ^14^C of CO_2_ in soil pores in permafrost-rich environments has been found to be older than in ecosystem respiration (37). In fact, an overlap between the observed average Δ^14^C_DIC_ in the Mackenzie River system and the Δ^14^C–CO_2 soil pore_ is evident (Fig. 3). Indeed, an emerging feature of permafrost carbon feedback is the one of “lateral permafrost carbon mobilization” wherein soil pore water happens to be a key variable in the generation and terrestrial emission/transport of greenhouse gases from thawing permafrost (14, 45–47). Currently, however, there is very limited information on attributes of soil pore water (for example, changes in soil pore water pH during lateral transport as in Fig. 2) in the Mackenzie River system and in the northern regions in general (12, 14, 37, 45–47, 53). A study of soil pore water characteristics in a transect of permafrost wetland in Greenland suggests a dominance of lateral advection transport process in the mobilization of CO_2_, providing evidence of acidification of the permafrost table linked to CO_2_ bubble ebullition (46). However, the proportion of the DIC formed from exchange with soil pore CO_2_ and the impact on the chemical equilibrium of the carbonate system for river systems remain unknown.

We can provide additional constraint on the possible composition of CO_2_ from biospheric organic carbon oxidation by using the river POC load. The river POC in the Mackenzie system is dominated by biospheric carbon from a mixture of plant detritus and degraded and aged organic matter from soil (70–90% of the POC), alongside rock organic carbon inputs (14, 18, 19). The biospheric POC has been derived eroded from a large spatial area across the catchments. The Δ^14^C and δ^13^C values of river POC (Fig. 3A) can therefore provide additional constraint on the composition of CO_2_ from biosphere organic carbon oxidation (see Table S3). Further research is warranted to better understand the chemical and isotopic characteristics of this organic matter source in the context of permafrost thaw and DIC in the Mackenzie River system.

**Fig. 5.**
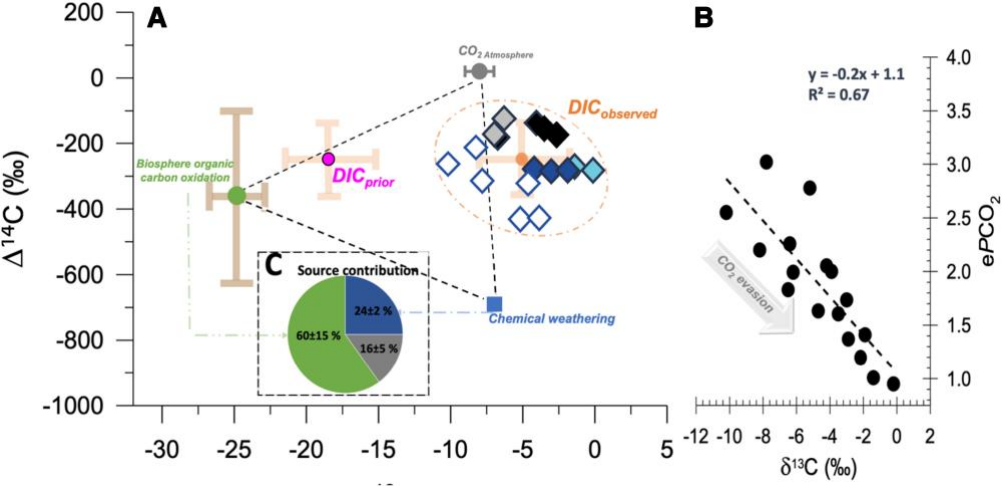
Role of CO_2_ evasion from the Mackenzie River system on DIC stable isotope composition. A) The mixing triangle is shown comprising of three likely sources of DIC in this river system (symbols as per Fig. 3). DIC_prior_ is estimated based on the expected maximum isotopic fractionation due to CO_2_ release. The “Biosphere organic carbon oxidation” isotopic endmember is a mix of river POC and CO_2 soil pore_ isotopic signatures shown in Fig. 3 with equal weightages given for both (see Discussion). B) The relation between e*P*CO_2_ is shown with the δ^13^C signature of DIC in this river system. The estimated uncertainty in calculation of the *P*CO_2_ is 10% similar to previous study (40). C) The relative source contributions to DIC_prior_ is shown.

Here, for this preliminary isotopic investigation of DIC from a northern river, we give equal weight to the dual-isotopic signatures of river POC (18) and CO_2_ measured in northern peatland soil pores (37) to define a varied biosphere organic carbon oxidation isotopic endmember. We report the endmember as mean ± SD (Fig. 3B; Table S3) of the available data from published works (18, 37). A two-component mixing (chemical weathering + biosphere organic carbon oxidation) could partly explain the observed Δ^14^C_DIC_ in the Mackenzie River system. However, the average Δ^14^C_DIC_ in the Mackenzie River system is less aged than this predicted two-component mixing (Fig. 3B). A three-component mixing including atmospheric CO_2_ (Δ^14^C = ∼20 ‰) could then explain the observed Δ^14^C_DIC_ values in the Mackenzie River system. While such a mixing is feasible, the observed dual-C isotopic composition of river DIC is mostly outside this predicted three-component mixing triangle (Fig. 5A). We hypothesize that this could potentially be linked to the stable carbon isotopic fractionation of DIC due to the outgassing of CO_2_ from the river surface (40, 43).

### A role for CO_2_ evasion from the Mackenzie River

To explain the observed δ^13^C_DIC_ values, we hypothesize that outgassing of CO_2_ is a key contributor. River CO_2_ in equilibrium with HCO_3_^−^ has lower δ^13^C_DIC_ values, meaning release of CO_2_ from river surfaces could drive the DIC pool to higher δ^13^C_DIC_ values. There are also potential fractionation effects at the water surface (39). To explore the role of CO_2_ evasion on the stable isotope composition, we calculate the excess *P*CO_2_ (e*P*CO_2_) in the river water, i.e. the ratio of *P*CO_2_ in the sample calculated from field-determined pH and temperature to that of the atmosphere. Measurement of pH during the sampling campaign allowed for the estimation of *P*CO_2_ of the water samples as conducted elsewhere (40). In general, CO_2_ diffuses out of waters when the *P*CO_2_ of the solution is greater than that of the ambient atmosphere (16, 32, 40). In the present dataset, the e*P*CO_2_ ranged between 1 and 3 (Fig. 5B) which is comparable with previously reported e*P*CO_2_ values from the Mackenzie River system (54). It has been found that the isotopic fractionation of DIC due to CO_2_ loss becomes significant for e*P*CO_2_ of ∼2 and above (55, 56). Indeed, we find that as e*P*CO_2_ decreases, the δ^13^C_DIC_ values increase (Fig. 5B). This suggests that loss of CO_2_ from river surfaces lowers e*P*CO_2_ and impacts δ^13^C_DIC­_, altering any primary source signal. Similar patterns have been linked to the evasion of CO_2_ in headwater catchment streams (40–43). Also, other studies at carbonate springs and in acidic headwater catchments have documented similar shifts in δ^13^C_DIC_ due to CO_2_ outgassing (56–58). However, the extent of the relationship between e*P*CO_2_ and isotopic fractionation of HCO_3_^−^ due to CO_2_ loss remains unknown for this river system.

In open systems, the kinetic isotopic fractionation associated with this process (ε_HCO3− CO2_) is estimated to be ∼14.7‰ (41), which is greater than the reported equilibrium value of ε_HCO3− CO2_ = 8‰ (59–61). Assuming this to be the case, we recalculate a minimum δ^13^C_DIC_ value prior to CO_2_ evasion in the Mackenzie River main stem of ∼−19 ± 3‰ (see “DIC_prior_” in Fig. 5). We can also assess the fractionation using the empirical data here (Fig. 5). If we use the highest reported e*P*CO_2_ ∼4 from the Mackenzie River system (54), the value for isotopic fractionation is estimated (based on the relationship in Fig. 5B) to be ∼−17‰. These two approaches thus return near similar estimates of stable isotope fractionation due to CO_2_ evasion in the river system. The ^14^C values undergo correction for isotope fractionation as part of the reporting of radiocarbon measurements (62) wherein Δ^14^C is “normalized” where the effect of fractionation is removed, as such changes should not occur in Δ^14^C during CO_2_ evasion. The release of CO_2_ could be linked to the formation of secondary carbonate minerals in rivers. However, many rivers around the world are over saturated with respect to carbonate precipitation (63), yet lack clear direct evidence for secondary river carbonates. This remains an open question for the Mackenzie River system, although we note that secondary carbonate precipitation coupled to CO_2_ evasion would lead to minimal net shift in the δ^13^C_DIC_ value of the total DIC pool.

The sources of DIC can be reassessed by considering our calculated δ^13^C_DIC_ value of DIC_prior_, assuming it has the same Δ^14^C as DIC_observed_. This correction now places the DIC_prior_ within the source mixing triangle previously described (Fig. 5A). The DIC_prior_ has shifted closer to the biosphere organic carbon oxidation endmember, which can further explain the presence of observed carbon ages in the Mackenzie River system. In other words, the CO_2_ inputs from respiration of organic matter mix are partly aged and mix with the old C sourced from carbonate weathering inputs. We also find a potential role for isotopic exchange with the atmosphere (Fig. 5A). A quantitative source apportionment of the DIC_prior_ using Bayesian statistical approach (see Materials and methods) suggests that biosphere organic carbon oxidation could contribute as much as 60 ± 10% to the DIC in this river system as a whole (Fig. 5C). Together, this implies that aged CO_2_ from the landscape is leaking from the Mackenzie River system. This is likely happening during DIC transit from the tributaries to the deltaic region (as witnessed in the decreasing radiocarbon age of DIC between the riverine tributaries and main channel). It therefore appears that large-scale mobilization of greenhouse gases from aged carbon pools in permafrost soils is viable through hydrologically connected waters and degradation of river organic matter pools.

### Wider implications

The aged carbon measured in the DIC pool of the Mackenzie River system can be explained, once accounting for stable isotope fractionation during CO_2_ release from rivers, as a mixture of carbonate weathering processes and biospheric organic carbon oxidation (Fig. 5A). The oxidation of aged organic matter represents a leak of carbon from millennial storage on land, and is a pathway of concern for future climate warming in permafrost zones (9). Streams and rivers may be a route of this old carbon out from deep soils, into an open system where *P*CO_2_ promotes river CO_2_ release (Fig. 5B). If the DIC from carbonate weathering is derived from sulfide oxidation, the carbon is a leak of geological carbon (7) and appears to be of similar magnitude to organic carbon oxidation in the river system (Fig. 5B). Enhanced sulfide oxidation coupled carbonate weathering and organic matter oxidation have both been linked to increase temperature (29, 30, 53).

To understand the role of river CO_2_ release in the modified biogeochemical cycles of the Arctic, we require more focus on the age and isotope composition of DIC. Seasonal and time-series ^14^C_DIC_ samples, in analogy to sampling efforts made for DOC and POC (19), are needed to shed light on how changing hydrological pathways are modifying carbon pathways to river systems (12–14). Alongside these samples it is necessary to better understand the CO_2_ release fluxes from river surfaces. Potential factors such as DIC delivery, *P*CO_2_ gradient, pH, and turbulence in the river likely drive river CO_2_ evasion (17, 28, 32, 40). Such information is currently limited for the Mackenzie River system, wherein measurements have been sparse and mostly concentrated at few locations, e.g. (54), and not along the transect of the river, e.g. (64). The role of secondary carbonate precipitation (63, 65) and its influence on the fluxes and isotopic composition of CO_2_ also remains unknown. Further investigation of concentrations, fluxes, and isotopic composition of greenhouse gases is therefore much warranted from this high Arctic river system and others, in order to better understand the drivers of greenhouse gas release in such climatically vulnerable northern frontiers.

## Materials and methods

### Sampling

River samples were collected in July 2013 and June 2017. The July 2013 samples are from high/receding water stage (Fig. S2), for the Mackenzie River at Tsiigehtchic, in the Delta (middle channel), the Peel River, Arctic Red River, and the tributaries of the Peel River (Fig. 1). In June 2017, the main sites (Mackenzie River at Tsiigehtchic and delta, the Peel River, Arctic Red River) were resampled at high river flow, shortly after ice breakup (Fig. S2). In order to assess any potential vertical variation, we used a modified horizontally mounted ∼5.1 L Niskin bottle to recover water from different depths (18). For DIC measurements, we followed the protocol of Bryant et al. (66). One-liter capacity foil bags (FlexFoil PLUS), composed of four layers (polypropylene, polyethylene, aluminum foil, and polyethylene), were adapted to allow easy introduction of liquid sample. River water was filtered directly into weighed foil bags through polysethersulfone filters (Ø 142 mm, 0.22 μm). Prior to sample collection, each foil bag was sample-rinsed by attaching the Tygon tubing to the Niskin sampler tap, removing the clip on the Tygon tubing and allowing approximately 50 mL of water to enter the bag, and then allowing the bag to drain. The foil bag was then filled to ∼200–500 mL depending on expected DIC concentrations (half the bag capacity) and then, held with the outlet pointing upwards, the bag was gently squeezed so that the Tygon tubing remained water-filled before reapplying the clip, to ensure no air was trapped in the sample bag. The filled bag was reweighed and refrigerated in the dark at 4 C during fieldwork, shipped to the UK and the sample was frozen within ∼1 week of collection. Aliquots for ion analysis were collected in acid-washed high-density polyethylene bottles following methods outlined in Horan et al. (27). Further sampling details can be found in Table S1.

### Measurements

Water-soluble ion measurements were carried out at Durham University in the UK using a Dionex Ion Chromatography system (DX-120, Thermoscientific) with an analytical reproducibility of 5%. Parameters such as water temperature and pH were measured onsite using handheld probes calibrated each field day (Hannah Instruments pHep). The storage and hydrolysis of the water samples for DIC concentration and isotopic measurements was based upon the method described elsewhere (66) and conducted at NEIF Radiocarbon Laboratory in East Kilbride, UK. Briefly, the cryogenic isolation of CO_2_ from the water sample is achieved by introducing orthophosphoric acid into the water sample transferred into a hydrolysis vessel (66). This CO_2_ is passed through two dry ice–ethanol cryogenic traps, followed by two liquid nitrogen traps to cryogenically isolate the evolved CO_2_. Pressure readings of the evolved CO_2_ provide the DIC concentration.

Stable carbon isotope measurements were carried out on an aliquot of the recovered CO_2_ using a dual-inlet stable isotope mass spectrometer (Thermo Fisher DELTA V Plus), calibrated with international standards and reported as δ^13^C ‰ relative to Vienna Pee Dee belemnite. A second aliquot of the recovered CO_2_ was converted to graphite by Fe/Zn reduction and measured for ^14^C content on an accelerator mass spectrometer (AMS; National Electrostatics Corporation, USA) at the SUERC AMS Laboratory. The ^14^C data are reported as Δ^14^C, i.e. as per mil deviation from the AD 1950 decay-corrected NBS oxalic acid standard (62). Further methodological details can be found elsewhere (67).

### Bayesian statistical source apportionment

By combining the dual isotope signatures (Δ^14^C and δ^13^C) and assuming mass balance, it is possible to explore the relative contributions from various sources using a forward modeling approach:


$$\begin{array}{rl} & \left\{\begin{array}{c} \Delta^{14}C_{\mathrm{sample}}\\ \delta^{13}C_{\mathrm{sample}}\\ 1 \end{array}\right\}=\left\{\begin{array}{ccc} \Delta^{14}C_{\mathrm{bio}.\;C\;\mathrm{oxdn}.}\; & \;\Delta^{14}C_{\mathrm{chem}.\;\mathrm{weath}.}\; & \;\Delta^{14}C_{\mathrm{atm}.\;C\;}\\ \delta^{13}C_{\mathrm{bio}.\;C\;\mathrm{oxdn}.}\; & \delta^{13}C_{\mathrm{chem}.\;\mathrm{weath}.}\; & \;\delta^{13}C_{\mathrm{atm}.\;C}\\ 1 & 1 & 1 \end{array}\right\}\\ & \;\;\;\;\times \left\{\begin{array}{c} \;f_{\mathrm{bio}.\;C\;\mathrm{oxdn}.}\\ \;f_{\mathrm{chem}.\;\mathrm{weath}.}\\ \;f_{\mathrm{atm}.\;C} \end{array}\right\} \end{array}$$

where *f* denotes the fractional contribution from a given source, sample denotes the value of the analyzed field sample and the other isotope-values are source signatures (“bio. C. oxdn.”, “chem. weath.” and “atm. C” corresponding to biosphere organic carbon oxidation, chemical weathering, and atmospheric input, respectively). Two main complexities exist for solving this forward mixing model. The first regards the variability in the isotopic signatures of Δ^14^C and δ^13^C of various source classes, i.e. endmember variability (e.g. Table S3). The uncertainties in endmembers dominate over the measurement uncertainties. It is recognized that in order to correctly estimate the relative source contributions and related uncertainties, the endmember variability as well as other sources of uncertainty needs to be included in the analysis. While the biospheric organic carbon end member could be highly variable (18), we conclude this mixing analysis is still worthwhile, to establish the potential input of this component. Markov chain Monte Carlo (MCMC)-driven Bayesian approaches have been implemented to account for multiple sources of uncertainties/variabilities (68, 69). The MCMC approach used here was developed in detail in Andersson et al. (70) and builds on Andersson (68); https://github.com/mskoldSU/Andersson_et_al_2015 (open-access R-code) and has been used in multiple atmospheric aerosol studies, e.g. (71–73) as well as for other systems such as the isotope-based source apportionment of polycyclic aromatic hydrocarbons in 34 soils and the isotope-based source apportionment of organic carbon in sediments (74, 75). The statistical treatment of the endmember variability (in this approach) is the same regardless if one separates liquid fossil vs. coal in black carbon aerosols or permafrost vs. plankton in marine sediments. The resulting probability density functions output from the model give a “least-biased” representation of the precision. As such, here we have estimated the relative contributions from three likely sources to riverine DIC in the Mackenzie River system based on this approach (Fig. 5C). The second complexity relates to processes which alter the stable isotope composition of DIC. As discussed in the main text, we provide a first-order correction for fractionation due to CO_2_ evasion and explore the resulting stable isotope composition with the mixing model. Future work that independently quantifies the CO_2_ evasion flux and its isotope composition, in addition to denser sampling in space and time, should remain a research priority.

## Supplementary Material

pgae134_Supplementary_Data

## Data Availability

All data are included in the manuscript and/or supplementary material.
