# Supplementary material for: Leakage of old carbon dioxide from a major river system in the Canadian Arctic
Source: PNAS Nexus. 2024 Mar 29;3(4):pgae134. doi: 10.1093/pnasnexus/pgae134 (PMC11010656; doi:10.1093/pnasnexus/pgae134)
Supplement: pgae134_Supplementary_Data [file pgae134_supplementary_data.pdf]

Supporting Information

Leakage of old carbon dioxide from a major river system in the Canadian Arctic

Sanjeev Dasari[1]. Mark H. Garnett[2]. Robert G. Hilton[1]*[1]

[1]Department of Earth Sciences. University of Oxford. Oxford. OX1 3AN. UK
[2]NEIF Radiocarbon Laboratory. SUERC. Rankine Avenue. East Kilbride. G75 0QF

*Corresponding Author: sanjeev.dasari@earth.ox.ac.uk; robert.hilton@earth.ox.ac.uk

Supporting information includes: 10 pages, 2 Figures and 4 tables

Contents

Supplementary Figures

Figure S1. The influence of chemical weathering on the Mackenzie River DIC age……………………………………S3

Figure S2. Average temperature and discharge characteristics of the Mackenzie River basin …………………………….S4

Supplementary Tables

Table S1. Sampling details of the Mackenzie River summer campaign of 2013 and 2017………….................................S5

Table S2. Isotope signatures of DIC collected during the Mackenzie River sampling in 2013 and 2017…………………S7

Table S3. Radiocarbon ($\Delta^{14}C$) and stable carbon ($\delta^{13}C$) endmember values for potential DIC sources……….……….S8

Table S4. Source contributions for predicted DIC in the Mackenzie River system ……………………………………….S9

References **(S10)**

Supplementary Figures

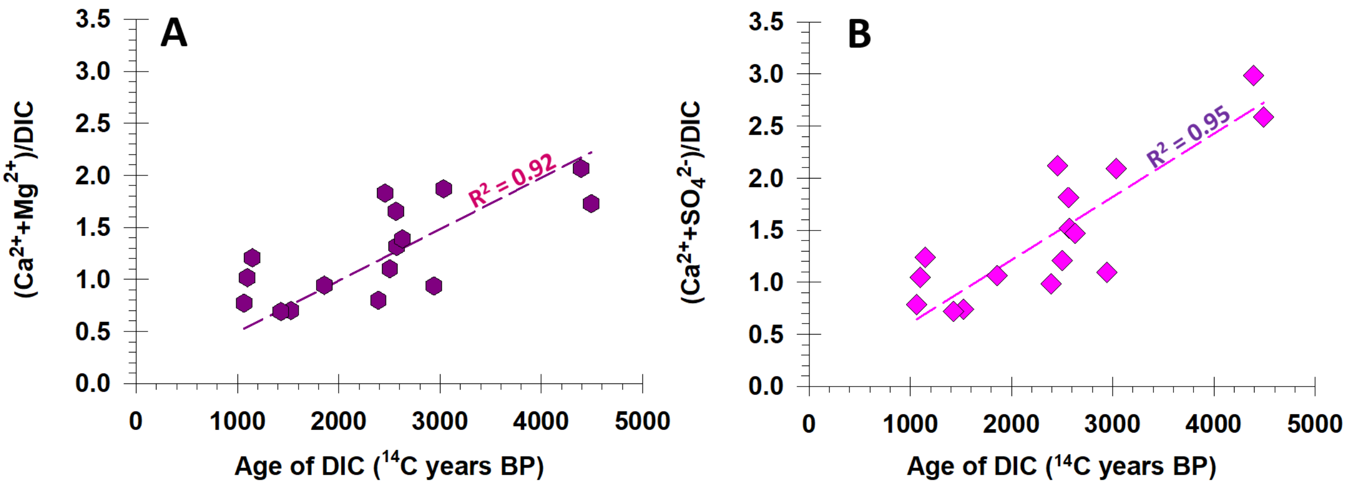

Figure S1. The influence of chemical weathering on the Mackenzie River DIC age.

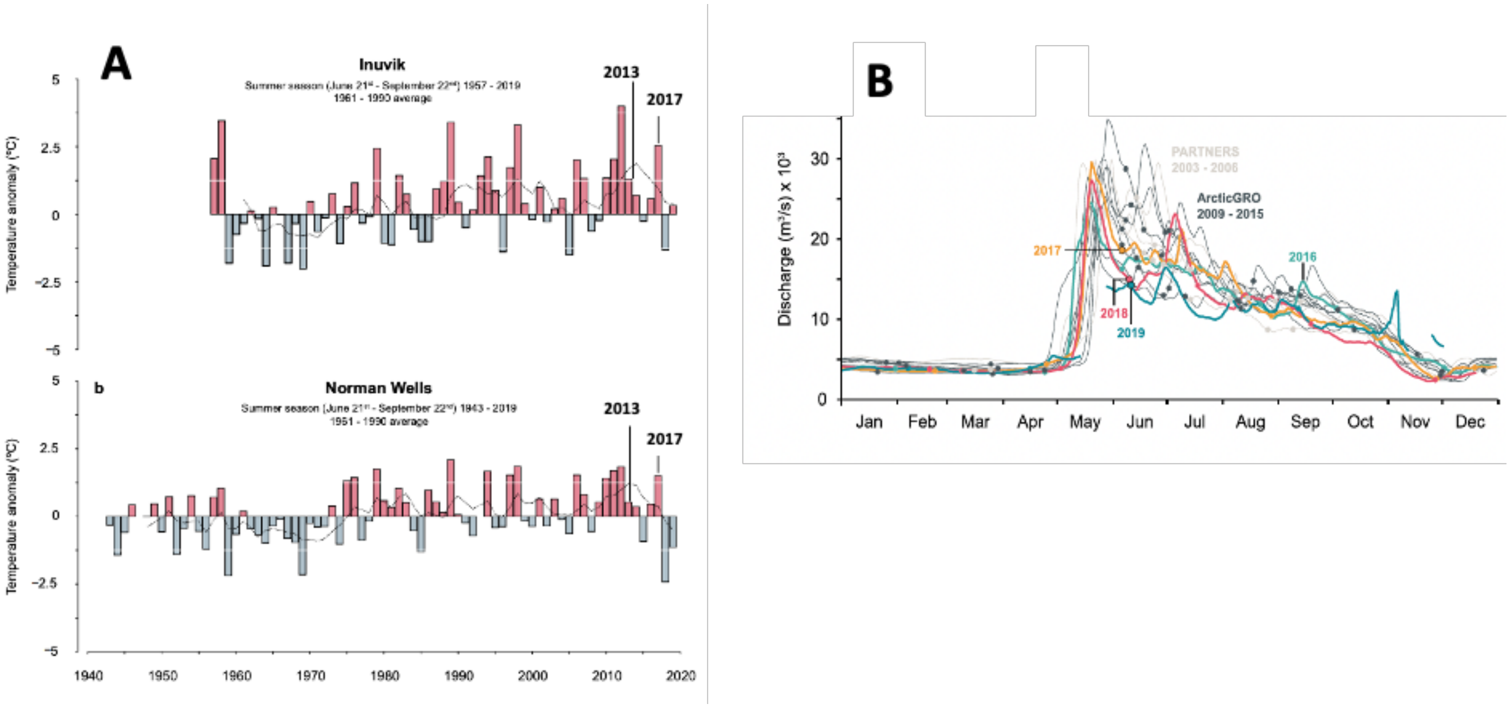

Figure S2. Average temperature and discharge characteristics of the Mackenzie River basin. (a) Average air temperature anomalies for the summer period from 1943 to 2019 from Inuvik research station. The baseline is defined as the mean over the 1961 - 1990 reference period. The summer seasons refers to the period from June 21st to September 22nd. The black line indicates a five-year mean (rolling average). Source: Environment Canada (http://climate.weather.gc.ca/). (b) Discharge at the Mackenzie River at Tsiigehtchic from 2003 to 2019 (http://www.wateroffice.ec.gc.ca). Dots denote sampling dates. The figures are reproduced from Schwab et al., 2020.

Supplementary Tables

Table S1. Sampling details of the Mackenzie River summer campaign of 2013 and 2017. The sampling locations and DIC, water soluble-ion concentrations are shown.

Sample ID	Sampling Date	Lat/Long	Basin	Location	pH	Temp (ºC)	DIC µmol/l	Mg^{2+} µmol/l	Ca^{2+} µmol/l	SO_4^{2-} µmol/l
CAN13-65	24/07/2013	68.41/134.09	Mackenzie	Middle Channel	8.05	17	1750	385	842	454
CAN17-06	03/06/2017	68.41/134.12	Mackenzie	Middle Channel	8.13	11	1072	367	843	412
CAN17-46	10/06/2017	68.41/134.12	Mackenzie	Middle Channel	8.40	13	1228	389	859	429
CAN17-53	10/06/2017	68.41/134.12	Mackenzie	Middle Channel	8.45	13	1041	394	863	425
CAN13-82	26/07/2013	67.45/133.73	Mackenzie	Tsiigehtchic			1833	398	868	451
CAN17-17	05/06/2017	67.45/133.73	Mackenzie	Tsiigehtchic	8.35	12	1538	364	824	384
CAN13-83	26/07/2013	67.45/133.73	Arctic Red	Tsiigehtchic			1500	759	1217	1058
CAN17-30	05/06/2017	67.43/133.78	Arctic Red	Tsiigehtchic	8.00	10	750	473	898	691
CAN13-81	26/07/2013	67.33/134.88	Peel	Fort Macpherson	8.00	11	1333	690	1162	797
CAN17-33	07/06/2017	67.32/134.87	Peel	Fort McPherson	8.40	11	1586	646	1101	816
CAN17-39	07/06/2017	67.32/134.87	Peel	Fort McPherson	8.40	11	1073	656	1118	830
CAN13-09	20/07/2013	65.71/137.99	Peel tributary	First Demptser sight	8.10	12	1250	777	1561	1052
CAN13-13	20/07/2013	65.35/123.29	Peel tributary	Dempster bridge	8.10	12	2167	1210	2534	3071
CAN13-25	22/07/2013	64.83/138.36	Peel tributary	Dempster bridge	7.75	11	1500	530	887	711
CAN13-30	22/07/2013	65.17/138.36	Peel tributary	Dempster	7.00	10	1417	902	2025	2203

CAN13-33	22/07/2013	65.36/138.30	Peel tributary	Dempster Bridge	7.80	14	1833	526	940	862
CAN13-45	23/07/2013	66.91/134.34	Peel tributary	Dempster	7.90	9	2750	691	1884	1124

Table S2. Isotope signatures of DIC collected during the Mackenzie River sampling in 2013 and 2017.

Sample ID	Basin	Accession #	F Modern (F_m)	F_m Error	$\delta^{13}C$ (‰)	$\Delta^{14}C$ (‰)	DIC age (^{14}C years BP)
CAN13-65	Mackenzie	SUERC-70361	0.83	0.0036	-6.4	-179.394	1527
CAN17-06	Mackenzie	SUERC-79785	0.85	0.0041	-2.1	-155.163	1293
CAN17-46	Mackenzie	SUERC-79795	0.87	0.0041	-3.5	-134.124	1096
CAN17-53	Mackenzie	SUERC-79796	0.87	0.0041	-2.9	-139.483	1146
CAN13-82	Mackenzie	SUERC-70363	0.84	0.0037	-6.5	-169.218	1428
CAN17-17	Mackenzie	SUERC-79786	0.88	0.0041	-6.2	-130.452	1062
CAN13-83	Arctic Red	SUERC-70364	0.73	0.0034	-0.2	-279.403	2571
CAN17-30	Arctic Red	SUERC-79787	0.74	0.0035	-1.4	-268.992	2455
CAN13-81	Peel	SUERC-70362	0.72	0.0033	-3	-284.499	2628
CAN17-33	Peel	SUERC-79788	0.73	0.0035	-4.2	-273.26	2503
CAN17-39	Peel	SUERC-79789	0.73	0.0035	-1.9	-275.542	2564
CAN13-09	Peel tributary	SUERC-70350	0.69	0.0032	-4.7	-319.678	3033
CAN13-13	Peel tributary	SUERC-70351	0.57	0.0027	-5.2	-434.328	4492
CAN13-25	Peel tributary	SUERC-70353	0.79	0.0035	-8.2	-212.324	1856
CAN13-30	Peel tributary	SUERC-70354	0.58	0.0027	-3.9	-425.492	4391
CAN13-33	Peel tributary	SUERC-70359	0.74	0.0034	-10.2	-262.891	2389
CAN13-45	Peel tributary	SUERC-70360	0.69	0.0032	-7.8	-311.756	2940

Table S3. Radiocarbon ($\Delta^{14}C$) and stable carbon ($\delta^{13}C$) endmember values for potential DIC sources. Note 'Biosphere organic carbon oxidation' isotopic endmember is a mix of POC_{river} and soil-pore CO_2 endmembers with equal weightage given to both sources. The references are marked beside each.

	Soil pore CO_2[1]	Ecosystem respiration[1]	POC_{river}[2]	DOC[3]	Biosphere Organic carbon oxidation	Silicate Weathering[4,5]	Carbonate Weathering (carbonic acid)[4,5]	Carbonate Weathering (Sulfuric acid)[4,5]	Rock Org. C[8]
$\Delta^{14}C$ [‰]	-143±90	+11±43	-578±175	-3±35	-360±250	-8	-500±50	-1000	-1000
$\delta^{13}C$ [‰]	-24±2	-26±1	-26±2	-24±6	-25±3	-17±7	-9±6	-1±1	-27±1

Table S4. Source contributions for predicted DIC in the Mackenzie River system. The $DIC_{predicted}$ isotopic signature is a combination of the proportional contributions[6-8] and the respective source endmembers in Table S3. Note $\Delta^{14}C$ and $\delta^{13}C$ of the $DIC_{predicted}$ is estimated to be -700±30 ‰ and -8±2 ‰, respectively.

**Estimated contributions to DIC**	**% HCO_3^- flux**
Carbonate weathering via carbonic acid	
i) (atmospheric CO_2: soil CO_2)	16
ii) $CaCO_3$ derived	24
Carbonate weathering via sulfuric acid ($CaCO_3$ derived)	38
Silicate weathering via carbonic acid (atmospheric CO_2: soil CO_2)	11
Rock organic carbon (CO_2 which supplies carbonic acid)	11

References

1. Vaughn, L.J. and Torn, M.S., 2018. Radiocarbon measurements of ecosystem respiration and soil pore-space CO2 in Utqiaġvik (Barrow), Alaska. *Earth System Science Data*, 10, 1943-1957.

2. Hilton, R. G., Galy, V., Gaillardet, J., Dellinger, M., Bryant, C., O'Regan, M., et al. 2015. Erosion of organic carbon in the Arctic as a geological carbon dioxide sink. Nature, 524, 84–87.

3. Schwab, M.S., Hilton, R.G., Raymond, P.A., Haghipour, N., Amos, E., Tank, S.E., Holmes, R.M., Tipper, E.T. and Eglinton, T.I., 2020. An abrupt aging of dissolved organic carbon in large Arctic rivers. *Geophysical Research Letters*, 47, GL088823.

4. Blattmann, T.M., Wang, S.L., Lupker, M., Märki, L., Haghipour, N., Wacker, L., Chung, L.H., Bernasconi, S.M., Plötze, M. and Eglinton, T.I., 2019. Sulphuric acid-mediated weathering on Taiwan buffers geological atmospheric carbon sinks. *Scientific reports*, 9, 2945.

5. Wang, W., Zhong, J., Li, S.L., Ulloa-Cedamanos, F., Xu, S., Chen, S., Lai, M. and Xu, S., 2023. Constraining the sources and cycling of dissolved inorganic carbon in an alpine river, eastern Qinghai-Tibet Plateau. *Science of The Total Environment*, 166262.

6. Gaillardet, J., Dupre´, B., Louvat, P., and Alle`gre, C. J., 1999, Global silicate weathering and CO2 consumption rates deduced from the chemistry of large rivers. *Chemical Geology*, 159, 3–30.

7. Calmels, D., Gaillardet, J., Brenot, A., and France-Lanord, C., 2007, Sustained sulfide oxidation by physical erosion processes in the Mackenzie River basin: Climatic perspectives. *Geology*, 35, 11, 1003–1006.

8. Horan, K., Hilton, R.G., Dellinger, M., Tipper, E., Galy, V., Calmels, D., Selby, D., Gaillardet, J., Ottley, C.J., Parsons, D.R. and Burton, K.W., 2019. Carbon dioxide emissions by rock organic carbon oxidation and the net geochemical carbon budget of the Mackenzie River Basin. *American Journal of Science*, 319, 473-499.
